# Parental knowledge, attitudes and beliefs regarding food allergy in the Netherlands – a cross-cultural comparison with the USA

**DOI:** 10.1186/2045-7022-3-S3-P145

**Published:** 2013-07-25

**Authors:** NJ Goossens, B Flokstra-de Blok, G vd Meulen, E Botjes, J Burgerhof, R Gupta, E Springston, B Smith, E Duiverman, A Dubois

**Affiliations:** 1 GRIAC Research Institute, University of Groningen, Groningen, the Netherlands; 2 Pediatric Allergy/Pulmonology, UMCG, Groningen, the Netherlands; 3 Department of General Practice, UMCG, Groningen, the Netherlands; 4 Pediatric Allergy, Martini Hospital, Groningen, the Netherlands; 5 Dutch Food Allergy Foundation, Nijkerk, the Netherlands; 6 Department of Epidemiology, UMCG, Groningen, the Netherlands; 7 Smith Child Health Research Program, Lurie Children’s Hospital, Chicago, IL, USA; 8 Institute for Healthcare Studies, Northwestern University Feinberg School of Medicine, Chicago, IL, USA; 9 Stitch School of Medicine, Loyola University of Chicago, Maywood, IL, USA; 10 Center for Management of Complex Chronic Care, Edward Hines Jr VA Hospital, Hines, IL, USA

## Background

Food allergic children are at least partially dependent on their parents to care for their food allergy. In addition, parents are often responsible for the education of others regarding food allergy, including the family, school, neighbours and friends. Aim of this study was to investigate food allergy knowledge, attitudes and beliefs of parents with food allergic children in the Netherlands. In addition, a cross-cultural comparison was made between parents from the USA and parents from the Netherlands.

## Methods

The original Chicago Food Allergy Research Survey for Parents of Children with Food Allergy (CFARS-PRNT) was translated into Dutch. Parents of children with at least one doctor-diagnosed food allergy were included. Knowledge scores and attitude/ beliefs scores were determined and compared to the data from 2945 parents from the USA. Predictors of overall knowledge scores were investigated.

## Results

299 Dutch parents of children completed the translated CFARS-PRNT. The mean overall knowledge score in the Netherlands was 9.9 after adjusting for guessing, compared to 12.7 in the USA (p<0.001). Attitudes and beliefs regarding food allergy among parents from the Netherlands were generally more optimistic than among parents from the USA. Twenty percent of the variance in overall knowledge scores (Netherlands and USA) could be predicted.

## Conclusion

Food allergy knowledge among parents of food allergic children from the Netherlands shows room for improvement. These parents tend to be more optimistic towards food allergy than parents from the USA. Several modifiable factors were identified which could predict food allergy knowledge of these parents. Such factors could be targets for interventions aimed at improving parental knowledge.

## Disclosure of interest

None declared.

